# Explainable machine learning predictions of dual-target compounds reveal characteristic structural features

**DOI:** 10.1038/s41598-021-01099-4

**Published:** 2021-11-03

**Authors:** Christian Feldmann, Maren Philipps, Jürgen Bajorath

**Affiliations:** Department of Life Science Informatics, B-IT, LIMES Program Unit Chemical Biology and Medicinal Chemistry, Rheinische Friedrich-Wilhelms-Universität, Friedrich-Hirzebruch-Allee 6, 53115 Bonn, Germany

**Keywords:** Cheminformatics, Drug discovery

## Abstract

Compounds with defined multi-target activity play an increasingly important role in drug discovery. Structural features that might be signatures of such compounds have mostly remained elusive thus far. We have explored the potential of explainable machine learning to uncover structural motifs that are characteristic of dual-target compounds. For a pharmacologically relevant target pair-based test system designed for our study, accurate prediction models were derived and the influence of molecular representation features of test compounds was quantified to explain the predictions. The analysis revealed small numbers of specific features whose presence in dual-target and absence in single-target compounds determined accurate predictions. These features formed coherent substructures in dual-target compounds. From computational analysis of specific feature contributions, structural motifs emerged that were confirmed to be signatures of different dual-target activities. Our findings demonstrate the ability of explainable machine learning to bridge between predictions and intuitive chemical analysis and reveal characteristic substructures of dual-target compounds.

## Introduction

Given the strong interest in artificial intelligence (AI), especially machine learning (ML) and deep learning, across chemical disciplines^1–3^ and the notorious “black-box” character of many ML models^4,5^, much attention is currently paid to explainable or interpretable AI^5–7^. Understanding predictions in chemical terms is often critical for the acceptance of computational modeling for experimental design. This also applies to the practice of medicinal chemistry where the discovery and further development of new active compounds represents the central task. These medicinal chemistry efforts have for long been supported by different types of compound activity predictions^3^. However, chemists are typically reluctant to synthesize newly predicted compounds if the predictions cannot be rationalized. In addition to predicting novel active compounds, ML might also be applied to better understand chemical characteristics of different types of compounds, as further discussed below. Such ML applications bridge between property predictions and explanatory or diagnostic modeling.

In medicinal chemistry and drug discovery, the concept of polypharmacology states that many active compounds and drugs elicit their therapeutic effects through interactions with multiple targets, for which experimental evidence has been mounting over the past decade^8–10^. During the post-phenotypic and molecular science-driven era of drug discovery, efforts have mostly concentrated on rendering active compounds as target-selective as possible. On the contrary, the notion of polypharmacology has triggered rising interests in generating multi-target compounds beyond serendipitous discovery^11–13^.

However, a prerequisite for designing such compounds is understanding how they might differ in structure and/or molecular properties from single-target compounds such that they become capable of specifically interacting with more than one target. In this context, ML can make important contributions. For example, if structural features exist that set multi-target compounds apart from corresponding single-target molecules, ML models trained on the basis of molecular structure representations should be able to distinguish between these compound classes. This was demonstrated for single- and multi-target compounds from medicinal chemistry^14^ and biological screening^15^, providing evidence for the existence of such structural features. Furthermore, ML models for many different target pairs were derived and shown to be highly predictive in distinguishing dual- from corresponding single-target compounds^16^. However, these models generally failed in cross-predictions on target pairs for which they were not derived, clearly indicating that distinguishing structural features were not a general signature of compounds with multi-target activity, but depended on individual targets and their combinations^16^.

Insights into the molecular basis of multi-target activities are indispensable for deriving design strategies for polypharmacological compounds. However, the studies referred to above only provided evidence for the existence of characteristic features of multi-target compounds, but did not reveal them. Accordingly, the next logical step should be the identification of those features that determine accurate predictions of single- vs. multi-target compounds. If such features can be identified, their potential chemical relevance can then be examined.

The feature identification task leads directly to approaches for ML model explanation. Interpretation methods applicable to ML activity predictions are for the most part model-dependent. Relevant approaches include feature weighting techniques for kernel-based or Bayesian classification models^17–19^, which reveal highly weighted representation features that strongly influence positive or negative predictions. Going beyond model-dependent approaches, explanatory methods that are generally applicable to ML models regardless of the algorithms that are used are principally preferred, but difficult to develop. As an ML model-independent technique, sensitivity analysis^20^ was applied to investigate the influence of feature value perturbations on activity predictions^21,22^. Accordingly, sensitivity analysis indirectly assesses feature relevance and becomes computationally demanding with increasing model dimensionality. As a different model-independent approach for a quantitative assessment of ML predictions, we have adopted the Shapley value (SV) concept^23^ from game theory^24^ that was originally developed to quantify contributions of individual players to the performance of a team^23,25^. In feature analysis and ML model interpretation, SVs quantify contributions of individual features of a given representation to a prediction outcome. For large features sets, the explicit calculation of SVs for all possible feature combinations also becomes computationally demanding. However, this limitation can be circumvented by deriving a local interpretation model for individual predictions that approximates the original ML model in corresponding regions of feature space. This approach is termed Shapley Additive exPlanations (SHAP)^26^ and can be conceptualized as an extension of the Local Interpretable Model-agnostic Explanations (LIME) approach^27^. For different compound activity prediction tasks, SHAP calculations made it possible to quantify the contributions of individual molecular features to a correct or incorrect prediction, independently of the complexity of an ML model^28,29^. Hence, SHAP is applicable to any ML algorithm including deep learning methods. Importantly, for decision tree methods, an algorithm for the exact calculation of local SVs has recently been introduced^30^. We have shown that SHAP and exactly determined local SVs strongly correlated in compound activity prediction for both tree-based classification and regression models (> 80%)^29^.

In this work, we have made a first attempt to systematically identify structural features that determine accurate ML predictions of dual-target compounds (DT-CPDs) vs. corresponding single-target compounds (ST-CPDs) and evaluated these features in chemical terms. For this purpose, exact local SV values were calculated and a global SV feature importance analysis scheme was devised. This approach was applied to DT- and ST-CPDs with activity against structurally and mechanistically unrelated targets (which best embody the polypharmacology paradigm) including monoamine oxidase B (MAOB)^31^, the A_2a_ adenosine receptor (A2aR)^32^, and acetylcholinesterase (AChE)^33^. All three proteins are popular pharmaceutical targets and implicated in various central nervous system (CNS) diseases, a major therapeutic area for polypharmacology. For target pairs including MAOB, ML models were derived that distinguished with high accuracy between DT- and ST-CPDs and features determining accurate predictions were identified and mapped onto compound structures. In DT-CPDs, features forming coherent substructures were driving their correct prediction and it was shown that the absence of these features in corresponding ST-CPDs was largely responsible for their correct prediction. Substructures identified by formal computational analysis represented confirmed signatures of different DT activities. The results presented herein should be of interest both from an ML and polypharmacology perspective.

## Results

### Study design

Data sets were assembled comprising DT-CPDs with activity against MAOB and A2aR (target pair 1), MAOB and AChE (target pair 2), and corresponding ST-CPDs (see “Methods” for details). Target pair-based data sets were prioritized because features determining predictions of multi-target compounds were previously shown to depend on specific target combinations^16^. The pairs were selected to share a target (MAOB), which made it possible to compare compounds with activity against related yet distinct target pairs (which did not share any DT-CPDs). Furthermore, the targets investigated herein were chosen for in-depth analysis because they belong to different protein classes with distinct ligand binding characteristics but are implicated in diseases falling into the same therapeutic area. Given these selection criteria, it is difficult to identify pairs of unrelated targets for which sufficient numbers of DT-CPDs have been reported. However, the pairs including MAOB met all criteria for our proof-of-principle investigation.

MAOB is an enzyme of the outer mitochondrial membrane that catalyzes the oxidative deamination of biogenic amines including neurotransmitters such as dopamine in the CNS^31^. A2aR is a G-protein coupled receptor and member of the adenosine receptor family involved in cAMP synthesis and a variety of intracellular signaling events^32^. AChE is a hydrolytic enzyme that degrades the neurotransmitter acetylcholine and is thus involved in CNS regulation^33^.

For each target pair, we constructed balanced random forest (BRF) classification models to distinguish between DT- and corresponding ST-CPDs (see “Methods”). The use of BRFs enabled the calculation of accurate local SVs for model explanation^30^. While the SV formalism is primarily applicable to explain individual predictions, we also performed global analysis of predictions by determining important features across different compounds. For accurately predicted DT- and ST-CPDs, representation features (i.e., layered atom environments; see “Methods”) were identified that were responsible for the predictions. These features were then mapped onto compounds to examine whether they formed meaningful substructures, leading to the detection of structural motifs characteristic of DT-CPDs.

**Figure 1:**
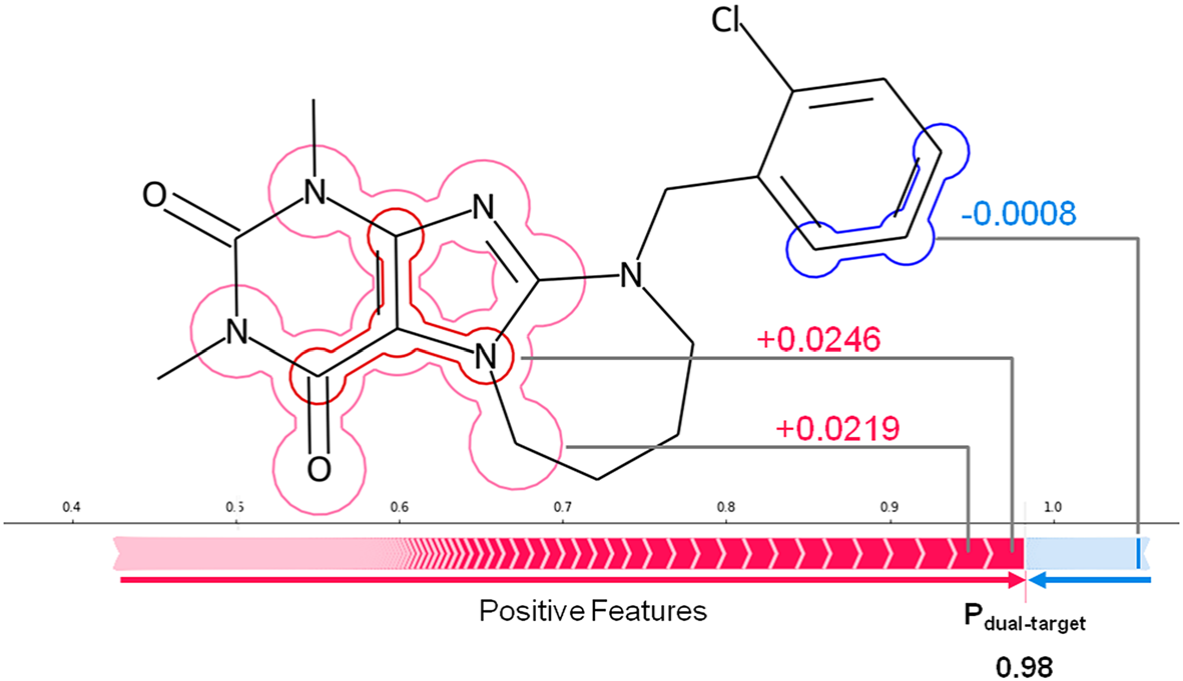
Shapley value analysis. For a test compound, positive (red) and negative (blue) SV feature contributions yield a probability *P* of DT activity. In this case, contributions from all but one feature present in the compound are positive. The sum of the base value of the classifier (0.5) and all feature importance values results in a probability of DT activity of 0.98.

Figure 1 illustrates SV-based feature importance assessment. Exact local SVs were calculated using the publicly available *Path Dependent Tree Explainer*^30^. For an individual compound, SV calculations yield quantitative feature contributions that support (positive SV) or oppose (negative) a given prediction. The sum of positive and negative contributions including the base value of the model (also termed expected value, representing the average SV for the training set) results in a class label probability (here probability of DT activity). Depending on the compound, different numbers of features might make positive or negative contributions of varying magnitude, as accounted for by SVs. Importantly, SV analysis also quantifies contributions of features that are absent in a compound to its prediction^28^. This ability is of critical relevance because the absence of specific features might also be responsible for a given prediction.

### Model performance

BRF models were trained on the basis of chemical structure only (see “Methods”). For each target pair, BRF models classified DT- and ST-CPDs with high accuracy (native predictions), as assessed on the basis of balanced accuracy (BA) and Matthews correlation coefficient (MCC) measures (see “Methods”) and reported in Table 1. Predictions over different trials were stable, yielding only very small standard deviations. Hence, ML consistently detected structural features distinguishing between DT- and corresponding ST-CPDs. As a control, BRF models derived for one target pair were applied to predict DT- and ST-CPDs of the other (cross predictions). In contrast to native predictions, these calculations failed, essentially yielding random accuracy, as also reported in Table 1. The control calculations confirmed that distinguishing structural features depended on each target pair and were not transferable. Highly accurate native predictions provided a meaningful basis for the quantitative feature analysis and model explanation.

**Table 1:** Accuracy of classification models.

Trained on	Tested on	Prediction task	BA	MCC
MAO B–A2aR	MAO B–A2aR	Native	0.95 ± 0.04	0.41 ± 0.03
MAO B–A2aR	MAO B–AChE	Cross	0.48 ± 0.02	− 0.02 ± 0.03
MAO B–AChE	MAO B–AChE	Native	0.90 ± 0.04	0.34 ± 0.04
MAO B–AChE	MAO B–A2aR	Cross	0.47 ± 0.01	− 0.02 ± 0.01

For native and cross predictions using BRF models, prediction accuracy is reported as the mean and standard deviation over 10 independent trials.

Table 2 reports the composition of the confusion matrix for training and test sets of single trials for both target pairs that are further discussed below. Only few DT-CPDs were misclassified. Due to high data imbalance, the number of false positives exceeded the number of true positives, which emphasizes the need to apply balanced performance measures such as BA and MCC.

**Table 2:** Elements of the confusion matrix for test and training sets.

	MAOB–A2aR		MAOB–AchE	
	Test set	Training set	Test set	Training set
True positives	23	26	31	35
False negatives	3	0	4	0
True negatives	2664	2675	1660	1688
False positives	85	74	164	134

### Shapley value-based feature exploration

Feature analysis aimed to identify features of the molecular representation (in this case, circular atom environments) that made large contributions to correct predictions and was carried out on the basis of individual models. Importantly, for compound classification, not only the presence, but also the absence of structural features is relevant. For example, frequent features in DT-CPDs that are not present in an ST-CPD might make strong contributions to its correct prediction (and vice versa). However, only features that are present in a compound can be analyzed within their structural/chemical context. Hence, features that are present in one (positive) compound class and absent in the other (negative) and whose respective presence and absence strongly contribute to correct predictions represent signature features of the positive class. The ability of SV analysis to quantify the contribution of features that are present or absent in a class sets the approach apart from other (model-dependent) feature weighting techniques.

### Feature contributions

**Figure 2:**
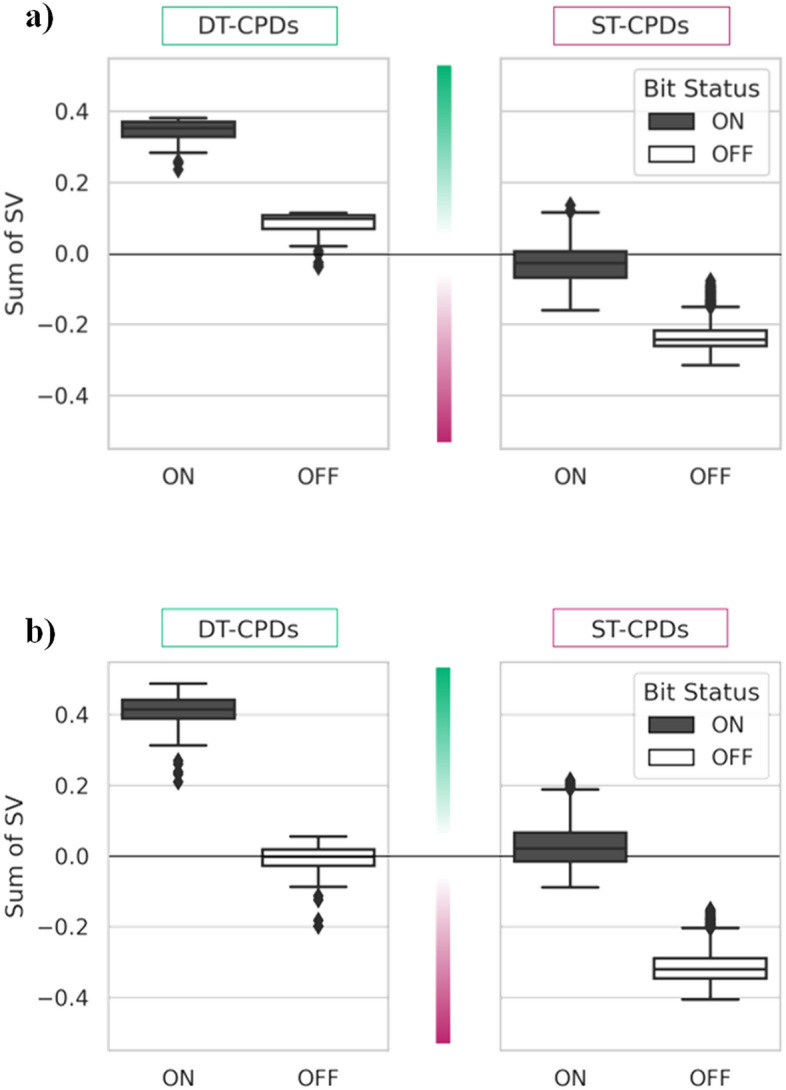
Global contributions of present and absent features. For each correctly classified DT- and ST-CPD, the sum of SVs was calculated separately for representation features that were present (bit status on, black) or absent (off, white). (**a**) Shows results for the MAOB-A2aR and (**b**) for the MAOB-AChE target pair. SV distributions are captured as box plots. The upper and lower whiskers indicate maximum and minimum values, the boundaries of the box represent the upper and lower quartiles, and the median is depicted as a horizontal line.

Test and training instances for both target pairs were subjected to global SV analysis. For each correctly predicted compound, the sum of SVs was calculated separately for features that were present or absent. The SV distributions for DT- and ST-CPDs in Fig. 2a indicate that correct predictions of DT-CPDs of the MAOB-A2aR target pair were largely determined by features present in these compounds, whereas predictions of corresponding ST-CPDs were mainly determined by features that were absent. Very similar observations were made for MAOB-AChE target pair compounds (Fig. 2b); a key result from global SV analysis. Thus, DT-CPDs shared features driving predictions, in contrast to ST-CPDs where the absence of these features was decisive for prediction accuracy.

### Feature extraction

**Figure 3:**
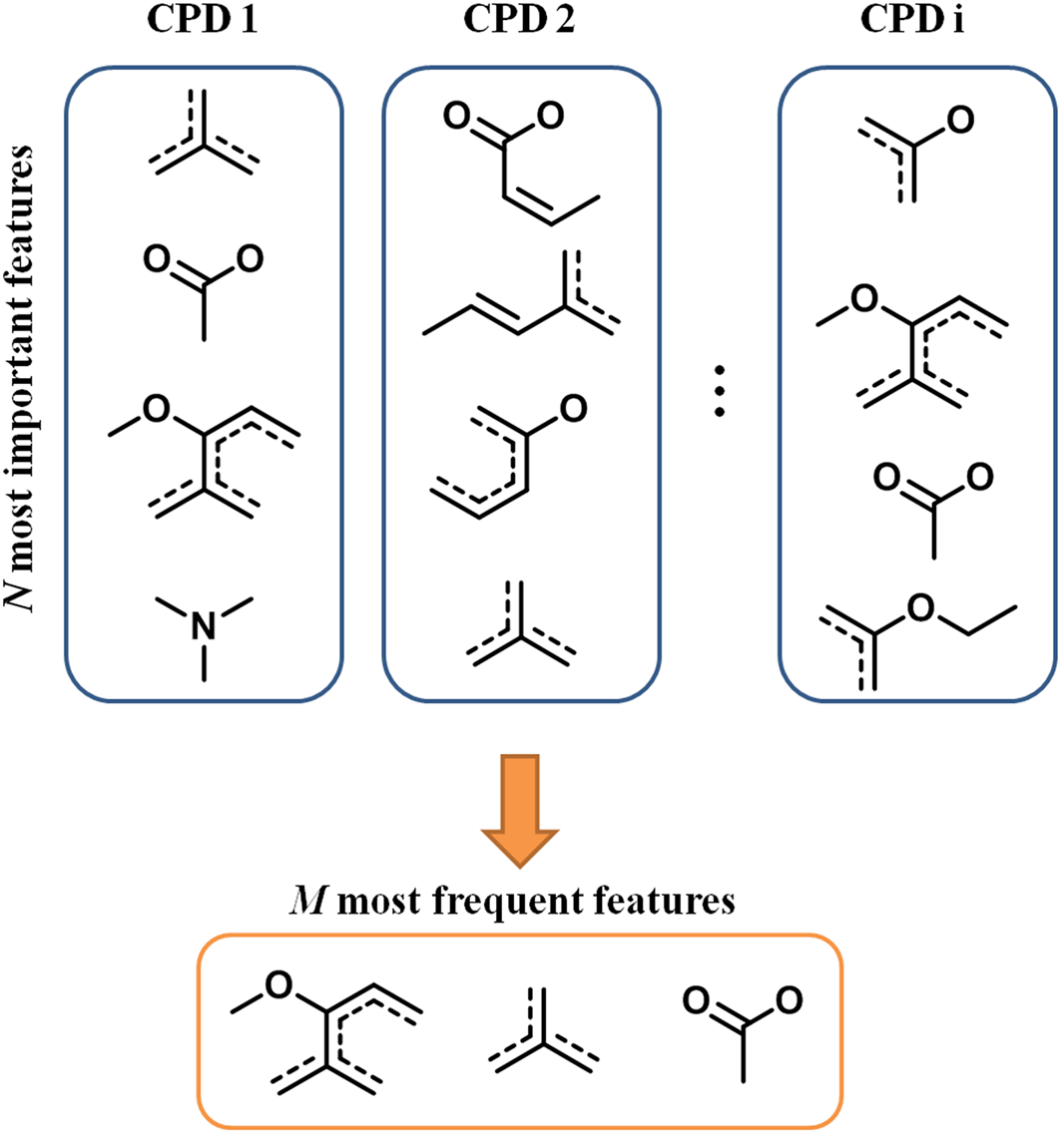
Feature extraction scheme. On the basis of SVs, the *N* most important features present in correctly predicted DT-CPDs were pre-selected and the *M* features occurring most frequently across these compounds were identified and prioritized.

On the basis of these findings, we next set out to identify features that were indicative of DT-CPDs. Therefore, the design of a feature extraction scheme was essential, taking into consideration that SVs of features represent contributions to individual predictions and thus differ from compound to compound. Hence, instead of applying a pre-defined threshold value for feature selection, which might only be met by a subset of correctly predicted compounds, features were extracted by rank. Therefore, the top *N* features with highest SVs were pre-selected from all features present in correctly predicted DT-CPDs. Then, each of these features was ranked by its occurrence across DT-CPDs, selecting the top most frequent *M* features. The approach is illustrated Fig. 3.

**Figure 4:**
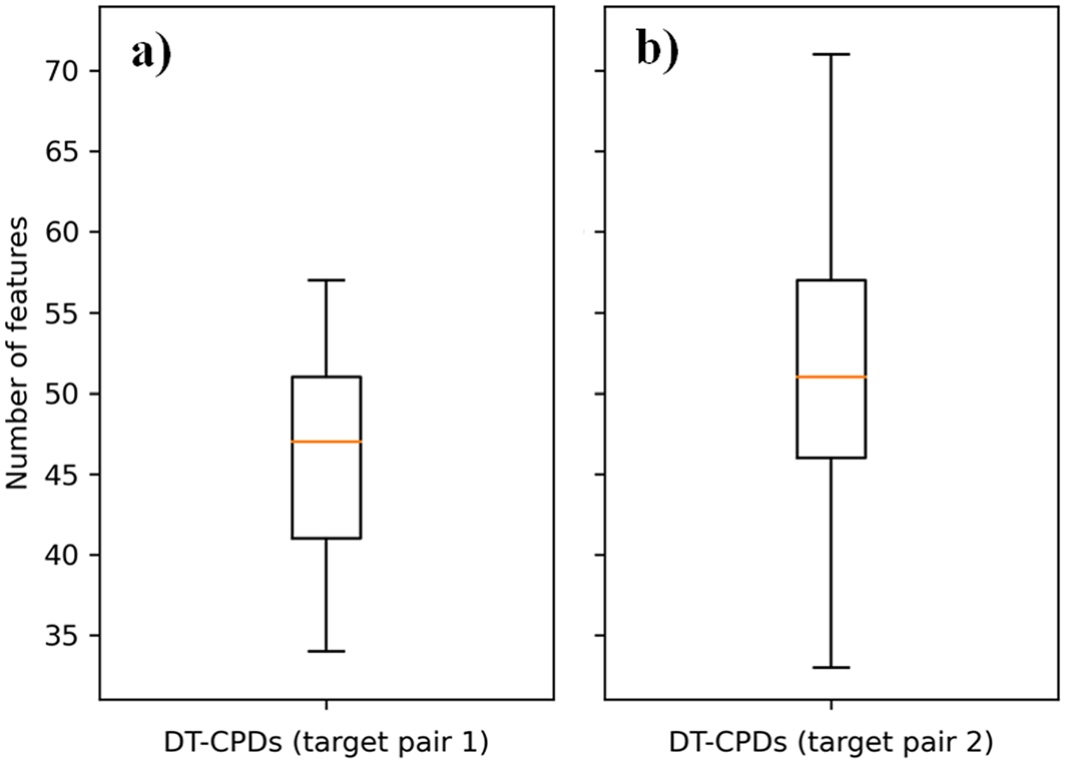
Feature distributions. Boxplots show the number of features per (**a**) MAOB-A2aR and (**b**) and MAOB-AChE DT-CPD.

For determining *N*, the number of features present across all DT-CPDs was considered. Figure 4a,b show that DT-CPDs for the MAOB-A2aR and MAOB-AChE target pair contained a median value of 47 and 51 features, respectively, and we thus pre-selected the *N* = 5 most important features with largest SV per compound (~ 10%). The top *N* = 5 features included 35 and 41 unique features for the MAOB-A2aR and MAOB-AChE target pair, respectively, from which the *M* = 10 most frequent features were prioritized for further analysis.

**Figure 5:**
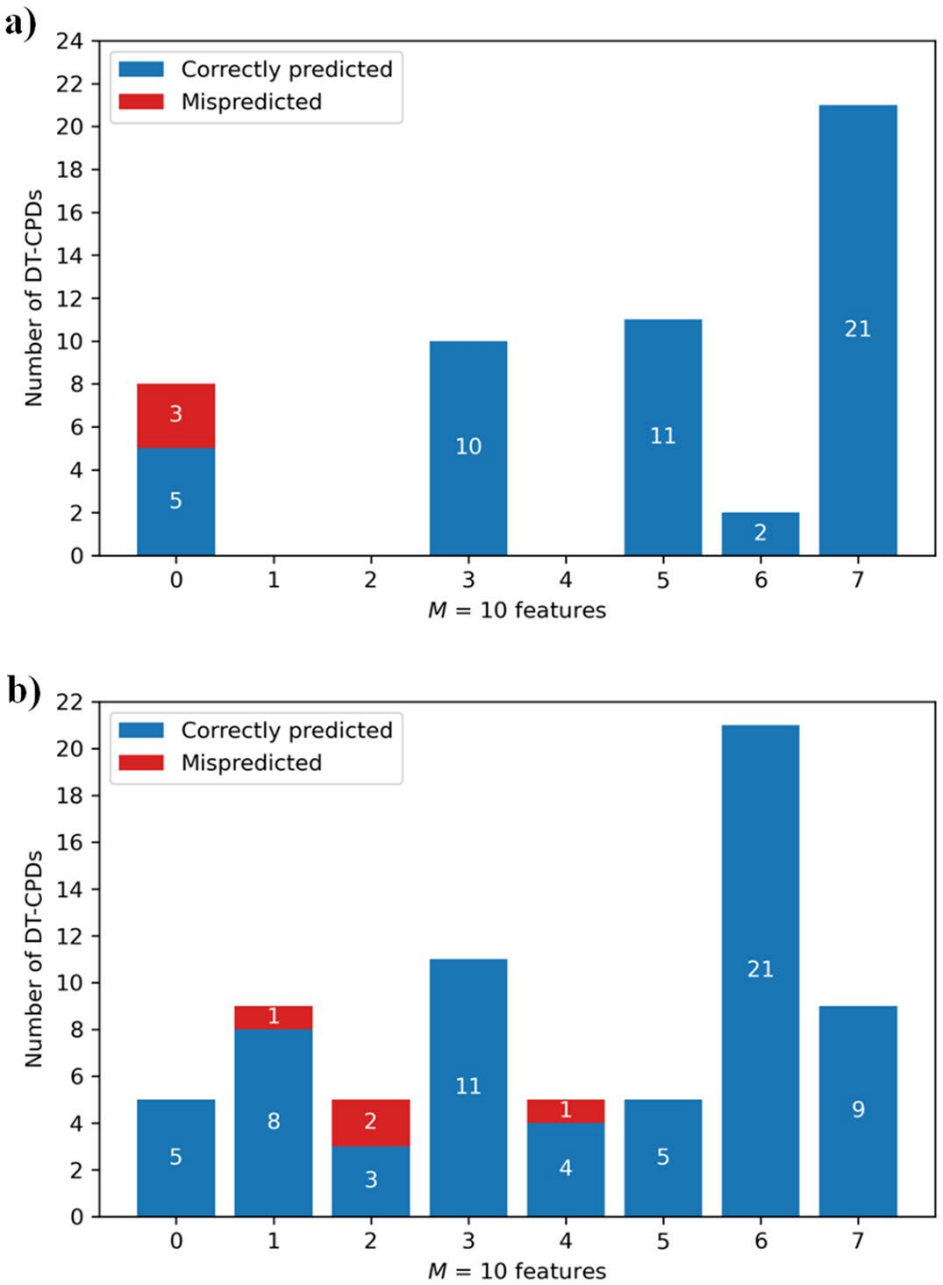
Distribution of prioritized features. The histogram reports the number of prioritized features in DT-CPDs for the (**a**) MAOB-A2aR and (**b**) MAOB-AChE target pair. Predictions are summarized for the two single trials reported in Table 2.

Figure 5a shows that only eight of the MAOB-A2aR DT-CPDs did not contain any of these features, three of which were incorrectly predicted in the single trial summarized in this figure. By contrast, each of the remaining training and test compounds contained at least three of the prioritized features (while no compound contained all 10 features) and all feature-containing compounds were correctly predicted. As shown in Fig. 5b, the feature distribution MAOB-A2aR DT-CPDs included the same feature range but differed in its details, as to be expected. Here, only five DT-CPDs did not contain prioritized features but were correctly predicted in the single trial. By contrast, four DT-CPDs with one, two, or four features were incorrectly predicted. As observed for the MAOB-A2aR target pair, the majority of DT-CPDs contained five to seven features and all of these compounds were correctly predicted for both target pairs.

### Mapping of features determining predictions

**Figure 6:**
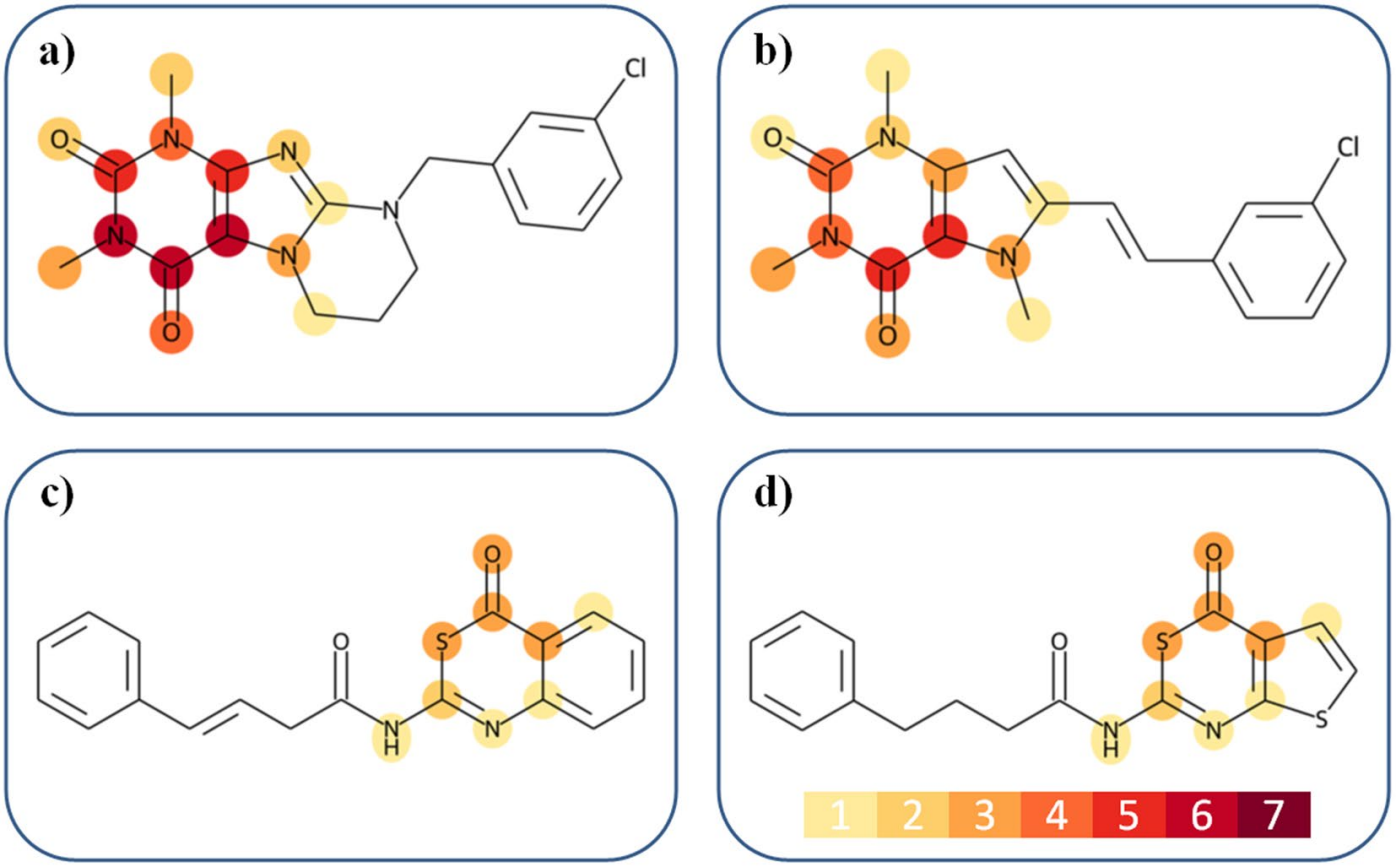
Feature mapping onto dual-target compounds from the first pair. Prioritized features are mapped onto to the structures of MAOB-A2aR DT-CPDs. Atoms are color-coded according to the number of features containing them, as indicated by the insert at the bottom of (**d**). Accordingly, the color code ranges from light yellow for atoms contained in one feature to dark red for atoms contained in seven features. Features determining the prediction of the compounds in (**a**) and (**b**) delineate a caffeine substructure while features contained in the compounds in (**c**) and (**d**) define a thiazine moiety.

Prioritized features making largest contributions to the correct prediction of MAOB-A2aR DT-CPDs were mapped on compound structures. Figure 6 shows four exemplary DT-CPDs with different numbers of most important features. The compound in Fig. 6a contains seven such features that delineate a caffeine substructure. The compound in Fig. 6b is a caffeine analogue in which a nitrogen atom is substituted by a carbon. It contains five prioritized features that also delineate the caffeine framework. Importantly, in both instances, features determining the correct prediction of DT-CPDs form a coherent substructure, thus indicating a moiety that might be characteristic of DT-CPDs, as further discussed below. However, this substructure is not an exclusive criterion for DT-activity, as illustrated by the compounds in Fig. 6c,d, which do not contain caffeine or an analogous substructure. Rather, the three prioritized features in these DT-CPDs outline the thiazine ring in the benzothiazine-4-one and the thienothiazine-4-one substructure, respectively. As a control, features absent in ST-CPDs that determined their correct prediction were also mapped to DT-CPDs. These features fully reproduced the caffeine moiety, but only marked the carbonyl group of the thiazine ring. Thus, high relevance of the caffeine substructure was emphasized by a larger number of prioritized features compared to thiazine whose absence was also of critical importance for the correct prediction of ST-CPDs.

**Figure 7:**
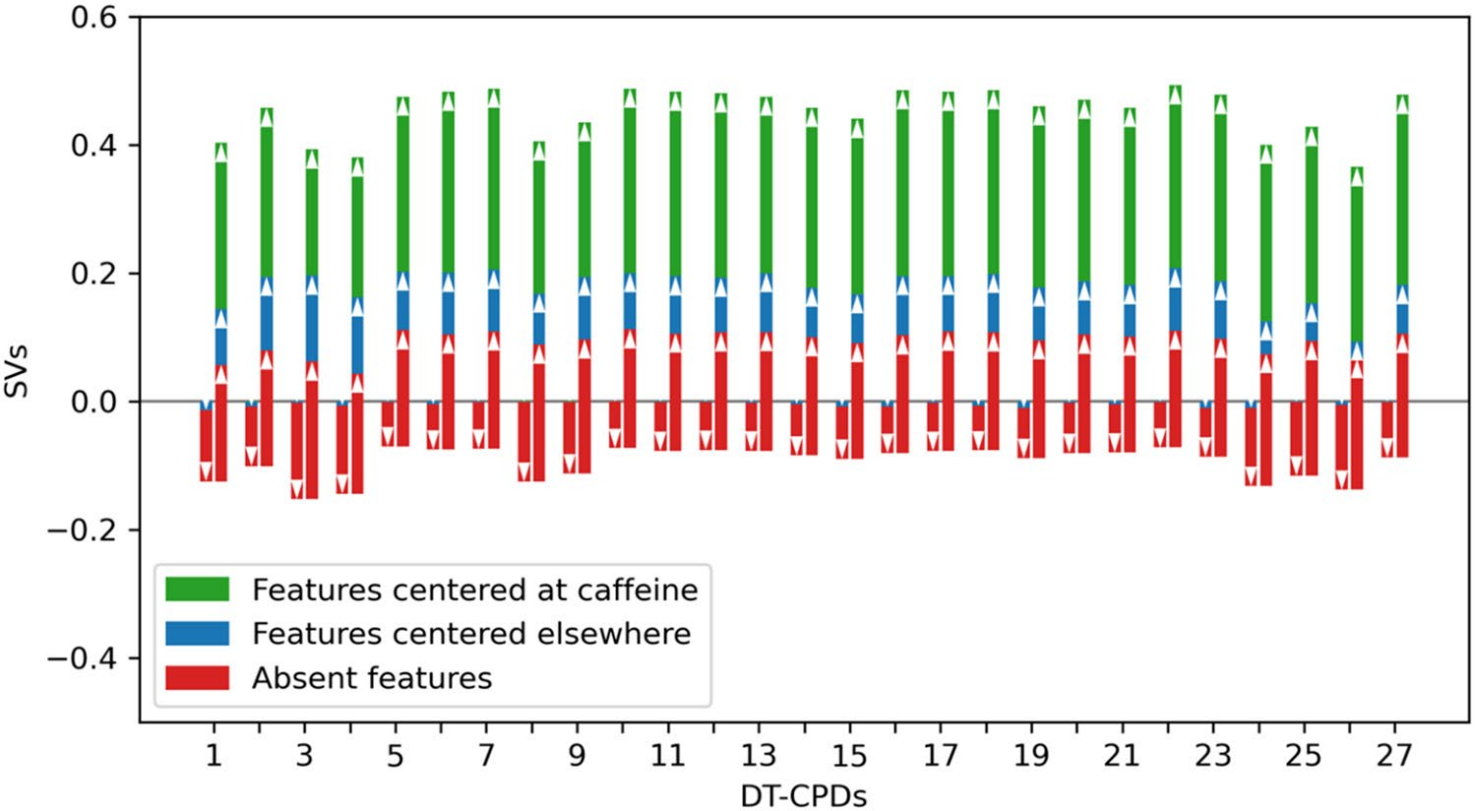
Contributions of caffeine-delineating features and others. For caffeine-containing DT-CPDs, cumulative SV contributions of features defining the caffeine moiety (green), features mapping elsewhere in the compound (blue), and absent features (red) are reported. The height of each bar accounts for the sum of feature SVs per compound.

In Fig. 7, contributions of features defining the caffeine substructure in DT-CPDs to predictions are compared to others. The magnitude of SV feature contributions is comparable across different compounds. Positive contributions of caffeine-related features were much larger than of features mapping to other parts of the structures. Furthermore, features absent in DT-CPDs either made positive or negative contributions of small magnitude, which essentially cancelled out. This was in contrast to ST-CPDs where the absence of caffeine-related features was often critically important for correct predictions.

The caffeine moiety was present in 27 of 52 DT-CPDs (51%) and found in only 135 of 5498 corresponding ST-CPDs (2%), nearly all of which were incorrectly classified as DT-CPDs. Many of these 135 putative ST-CPDs may not have been tested against both targets and hence represent false negatives due to data incompleteness. For 17 of these compounds, we found literature records of weak activity against the second target of the MAOB-A2aR (falling below our 10 µM potency threshold for compound selection; see “Methods”).

The purine ring contained in the caffeine substructure was found in 16% of correctly predicted ST-CPDs in different structural contexts distinct from caffeine, thus further emphasizing the critical relevance of the more specific caffeine substructure for accurate prediction of MAOB-A2aR DT-CPDs.

### Specificity of features

**Figure 8:**
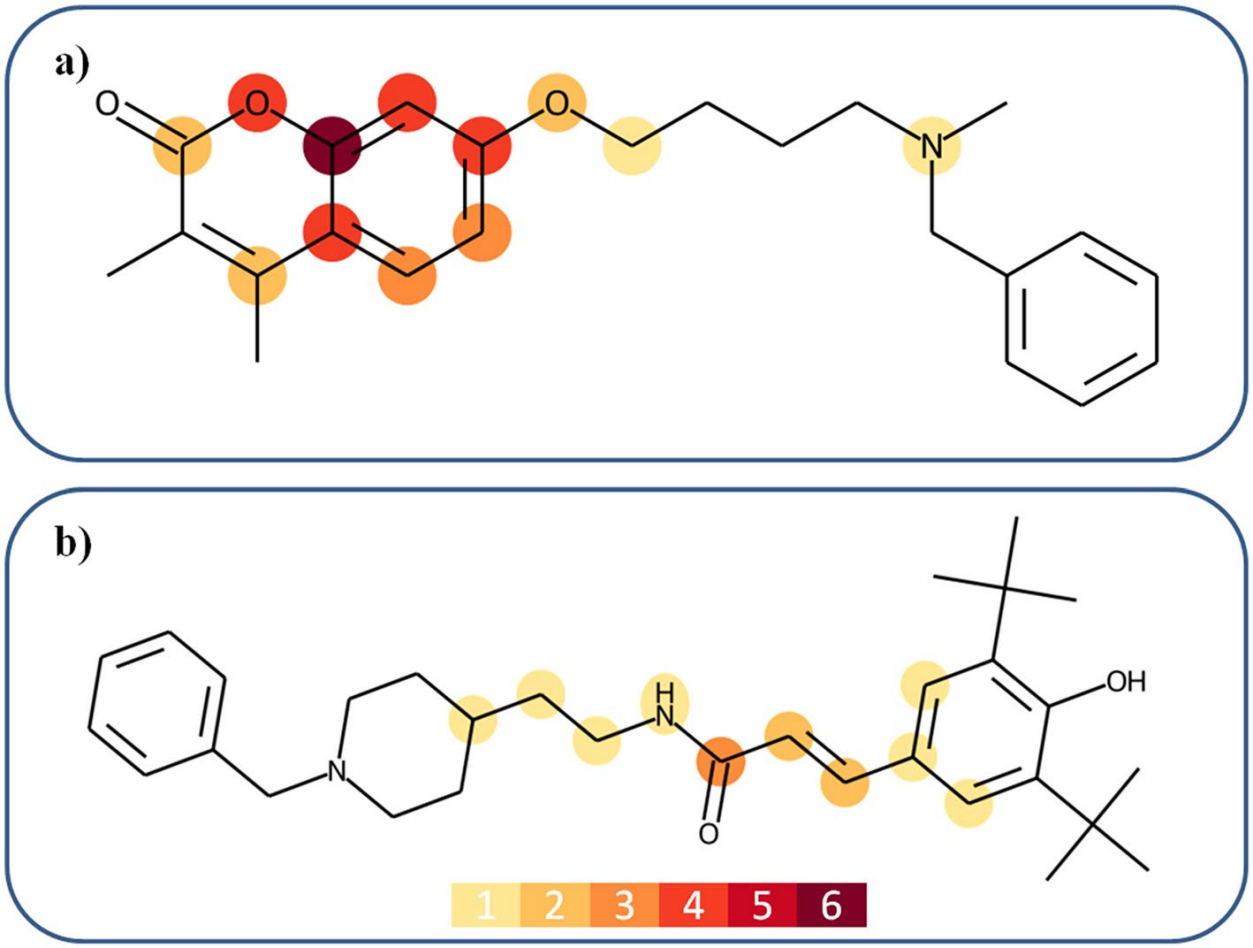
Feature mapping onto dual-target compounds from the second pair. Prioritized features are mapped onto MAOB-AChE DT-CPDs. The representation is according to Fig. 6. Atoms are color-coded according to the number of features containing them, as indicated by the insert at the bottom of (**b**). Accordingly, the color code ranges from light yellow for atoms contained in one feature to dark red for atoms contained in six features. Features determining the prediction of the compounds in (**a**) and (**b**) mostly delineate a coumarin substructure and an acrylamide linker fragment, respectively.

The analysis of feature importance also revealed prioritized features determining the prediction of DT-CPDs for the MAOB-AchE target pair. Moreover, most important features were found to delineate another specific coherent substructure in DT-CPDs, i.e., an ether-substituted coumarin depicted on the left in Fig. 8a. This coumarin substructure was present in 32 DT-CPDs (45%). In non-coumarin DT-CPDs, prioritized features preferentially encompassed an acrylamide connecting two aromatic and aliphatic ring systems (Fig. 8b). However, corresponding to the caffeine substructure for the MAOB-A2aR target pair, the coumarin moiety dominated correct predictions of MAOB-AchE DT-CPDs. For coumarin-containing DT-CPDs, cumulative SV contributions were similar to those observed for caffeine-containing DT-CPDs from the other target pair in Fig. 7.

The results showed that for DT-CPDs of two different target pairs containing MAOB, i.e., target pairs with overlapping yet distinct activity, features responsible for high prediction accuracy differed and defined distinct structural motifs; an interesting finding of our analysis, as further discussed below.

### From predictions to characteristic substructures

It must be emphasized that the model explanation analysis presented herein identified features that determined correct predictions of DT-CPDs. Of course, from a polypharmacology perspective, the ultimate goal is the identification of structural signatures of DT-CPDs that set them apart from corresponding ST-CPDs and can be used to guide compound design. The question is whether or not ML and in-depth analysis of predictions can aid in this process. As shown herein, features driving accurate predictions formed coherent substructures in DT-CPDs, hence providing an attractive basis for follow-up analysis in medicinal chemistry. At least in this case, the substructures identified by formal computational analysis indeed represented characteristics of different DT-CPDs. Literature searches confirmed that caffeine derivatives are active against both MAOB and A2aR^34^ and, in addition, coumarin derivatives active against MAOB and AchE^35^.

## Methods

### Target selection

Distinct targets from different families and classes were selected following the UniProt^36^ and Gene Ontology^37^ classification schemes. Target pairs were also required to share at least 50 DT-CPDs (see below). MAOB (UniProt ID: P27338), A2aR (P29274), and AChE (P22303) were selected, yielding two overlapping target pairs (MAOB-A2aR and MAOB-AChE). These targets are relevant for polypharmacology-oriented drug discovery since they are implicated in CNS pathologies such Alzheimer’s or Parkinson’s disease.

### Compounds and activity data

Compounds with a molecular weight of less than 1000 Da and their bioactivity data were obtained from ChEMBL (version 28)^38^. Only compounds with direct interactions (target relationship type: “D”) with human targets at the highest confidence level (target confidence score: 9) were considered. In addition, standard potency measurements (K_i_ IC_50_, and K_d_) with an exact value (“=”) were required (and recorded as negative decadic logarithmic values). Measurements flagged as “inactive”, “not active”, “inconclusive”, or “potential transcription error” were disregarded. In addition, weakly potent (borderline active) compounds (less than 10 µM) and potential assay interference compounds were removed using public filters^39–41^ to avoid potential false positive activity annotations.

On the basis of these data curation criteria, 52 DT-CPDs were obtained for the MAOB-A2aR target pair and 70 DT-CPDs for the MAOB-AChE pair. As expected, only limited numbers of DT-CPDs with high-confidence activity annotations were available. For first target pair, 1932 and 3566 ST-CPDs were obtained for MAOB and A2aR, respectively, and for the second target pair, 1793 ST-CPDs for MAOB and 1853 ST-CPDs for AChE. The number of ST-CPDs for MAOB differed for these pairs because small numbers of compounds active against MOAB with additional reported activities against targets related to A2aR or AChE were not selected.

### Molecular representation

For ML, compounds were encoded as a binary feature vector using the public RDKit^39^ implementation of the Morgan fingerprint with bond radius of 2^42^, which consists of molecule-specific layered atom environments (neighbor atom connectivity paths up to a bond radius of 2 for each atom in a compound). Hashed features were mapped to unique vector positions to avoid bit collisions. For feature analysis, this representation is principally preferred because recorded atom environments are partly overlapping, which provides a control for feature consistency of feature importance (i.e., mapping of prioritized overlapping features typically delineates coherent substructures in test compounds). Given our focus on structural characteristics of DT-CPDs, we did not consider simple physicochemical or other numerical descriptors for ML model building.

### Balanced random forest calculations

For compound classification, BRF models were derived. BRF is a supervised ML algorithm based upon an ensemble of decision trees, in which each tree is trained using an independently selected bootstrap sample from the training set^43,44^. Training samples selected for each tree are adjusted for class imbalance by randomly under-sampling the majority class; an important methodological aspect, given the intrinsic imbalance of DT- vs. ST-CPDs. Estimates of class probabilities for predicted instances are calculated as the mean class probabilities over individual trees. In decision trees, the probability for a class is equal to the fraction of samples of the given class in the final leave node. Optimal hyperparameters such as number of decision trees ('n_estimators': 25, 50, 100, 200, 400), minimal number of samples for a split ('min_samples_split': 2, 3, 5, 10), and minimum number of samples for a leave-node ('min_samples_leaf': 1, 2, 5, 10) were determined via  10-fold training set internal cross validation. The best performing hyperparameter combination was then used to generate the final classifier was trained with the complete training set. For predictions, the remaining DT- and ST-CPDs were used as test instances. Model performances were estimated as the average over 10 independent trials. As performance measures, BA^45^ and MCC^46^ values were calculated:

$${\text{BA}} = \frac{1}{2}\left( {{\text{TPR}} + {\text{TNR}}} \right)$$

$${\text{MCC}} = \frac{{{\text{TP}} \times {\text{TN}} - {\text{FP}} \times {\text{FN}}}}{{\sqrt {\left( {{\text{TP}} + {\text{FP}}} \right)\left( {{\text{TP}} + {\text{FN}}} \right)\left( {{\text{TN}} + {\text{FP}}} \right)\left( {{\text{TN}} + {\text{FN}}} \right)} }}$$

TP, TN, FP, and FN stand for true positives, true negatives, false positives, and false negatives, respectively.

## Conclusion

In polypharmacology-oriented drug discovery, structural motifs that are signatures of compounds with well-defined activity against more than one target are of prime interest. However, only little is currently known about structural features determining multi-target activity, which hinders the implementation of compound design strategies for polypharmacology. ML studies have provided evidence that structural features differentiating DT- and corresponding ST-CPDs exist and that they depend on given target combinations. However, the nature of such structural features has thus far remained largely elusive. We have reasoned that the identification of molecular representation features that determine ML predictions of DT- vs. ST-CPDs provides a basis for the exploration of structural characteristics of DT-CPDs. Therefore, we have devised a pharmacologically relevant overlapping target pair-based test system for a proof-of-concept investigation reported herein. Highly accurate ML prediction models were derived for this test system enabling an in-depth analysis of successful predictions. The SV concept from game theory was used for model explanation and extended for global feature analysis. This made it possible to quantify the influence of representation features that were either present or absent in test compounds, a unique aspect of the analysis concept, and identify most important features determining correct predictions. Quantifying the relative influence of feature presence and absence revealed small numbers of features whose presence in DT-CPDs and absence in corresponding ST-CPDs was decisive for accurate predictions, thus providing a possible rationale for DT activity. These features were specific for overlapping yet distinct DT activities. Moreover, they formed coherent substructures in DT-CPDs. Two structural motifs, caffeine and coumarin fragments, emerged that largely determined accurate predictions of DT-CPDs and that were both confirmed to represent characteristic substructures conferring different DT activities.

The analysis generally depends on the availability of compounds with known activity and ST- or DT-CPDs might also be active against additional targets for which no experimental data are currently available. However, this does not limit the potential of the approach because it is applicable to any ML models that accurately classify compounds according to known activity. If compounds with different activity profiles can be correctly classified, structural features driving the predictions can likely be identified and interpreted. If additional targets emerge for designated ST- or DT-CPDs, new prediction tasks can be formulated.

Taken together, the results of our study establish proof-of-concept for the ability of explainable ML to progress from the analysis of predictions to the identification of chemically relevant characteristics of DT-CPDs. The analysis scheme is fully reproducible and applicable to other target combinations and compound features.

## Data Availability

All calculations were carried out with public domain data and programs.
